# Cocaine Modulates the Expression of Opioid Receptors and miR-let-7d in Zebrafish Embryos

**DOI:** 10.1371/journal.pone.0050885

**Published:** 2012-11-30

**Authors:** Roger López-Bellido, Katherine Barreto-Valer, Fátima Macho Sánchez-Simón, Raquel E. Rodríguez

**Affiliations:** Department of Biochemistry and Molecular Biology, Institute of Neuroscience of Castilla y León, University of Salamanca, Salamanca, Spain; Rutgers University, United States of America

## Abstract

Prenatal exposure to cocaine, in mammals, has been shown to interfere with the expression of opioid receptors, which can have repercussions in its activity. Likewise, microRNAs, such as let-7, have been shown to regulate the expression of opioid receptors and hence their functions in mammals and in vitro experiments. In light of this, using the zebrafish embryos as a model our aim here was to evaluate the actions of cocaine in the expression of opioid receptors and let-7d miRNA during embryogenesis. In order to determine the effects produced by cocaine on the opioid receptors (*zfmor*, *zfdor1* and *zfdor2*) and let-7d miRNA (*dre-let-7d*) and its precursors (*dre-let-7d-1* and *dre-let-7d-2*), embryos were exposed to 1.5 µM cocaine hydrochloride (HCl). Our results revealed that cocaine upregulated *dre-let-7d* and its precursors, and also increased the expression of *zfmor*, *zfdor1* and *zfdor2* during early developmental stages and decreased them in late embryonic stages. The changes observed in the expression of opioid receptors might occur through *dre-let-7d*, since DNA sequences and the morpholinos of opioid receptors microinjections altered the expression of *dre-let-7d* and its precursors. Likewise, opioid receptors and *dre-let-7d* showed similar distributions in the central nervous system (CNS) and at the periphery, pointing to a possible interrelationship between them.

In conclusion, the silencing and overexpression of opioid receptors altered the expression of *dre-let-7d*, which points to the notion that cocaine via *dre-let-7* can modulate the expression of opioid receptors. Our study provides new insights into the actions of cocaine during zebrafish embryogenesis, indicating a role of miRNAs, let-7d, in development and its relationship with gene expression of opioid receptors, related to pain and addiction process.

## Introduction

Opioid effects are mediated by the μ-, δ-, and κ-opioid receptors (MORs, DORs, and KORs, respectively) [1]. The main effects of opioids - analgesia, tolerance and addiction- are essentially mediated by MORs [1], [2], while DOR agonists, in comparison with MORs, produce weak analgesic effects [3], [4]. Additionally, they do not bring about analgesic tolerance to morphine [4]. Opioid drugs, such as morphine and fentanyl, produce potent analgesic effects in comparison with other painkillers (e.g., nonsteroidal anti-inflammatory drugs), and hence they are widely used in the clinical management of pain, despite the adverse effects produced by chronic use, such as tolerance, physical dependence and addiction [5], [6]. When opioids are used over long periods of time, tolerance reduces the analgesic efficiency, and the opioid doses used must be raised for adequate levels of analgesia to be reached; this involves the risk of developing dependence and addiction to opioids [7], [8]. These adverse effects of opioids noticeably affect their optimal use in the clinical treatment of chronic pain conditions [9].

In human cocaine abusers, treatment with naltrexone (an opioid-antagonist) reduces the euphoria and the ‘crash’ produced by intravenous cocaine injection [10]. Also, administered systematically (in mammals) naloxone blocks both the reinforcing and conditioned motivational effects of cocaine [11], [12]. Likewise, an increase in β-endorphin (an endogenous opioid peptide) levels induced by acute treatment with cocaine has been found in the nucleus accumbens (NAc) [13], suggesting the possibility that the opioid system (opioid peptides and receptors) might be essential for the rewarding effects of cocaine. Moreover, other studies have shown that cocaine alters opioid receptor density in regions related to the addiction and reward circuits (in the nucleus accumbens and striatum) [14].

**Table 1:** Oligonucleotide primers employed.

Gen	Forward		Ta (°C)	Amplicon
*ef1α*	GTACTTCTCAGGCTGACTGTG	ACGATCAGCTGTTTCACTCC	55	136
*Zfmor*	ACGAGCTGTGCA AGATTGTG	CCGATTGCAGATGAAAGGAT	55	187
*zfdor1*	ACTATGAGAG CGTGGACCGTT	GCGGAGGAGAGGATCCAGAT	55	116
*zfdor2*	TCAGG CAAAACAATCTGCATG	CAGGATCATCAGGCCGTAGC	55	138
*dre-let-7d-1*	CGGTGTGAGGTAGTTGGTTGTAT	Universal qPCR Primer*	57	
*dre-let-7d-2*	GTAGTTGGTTGTATGGTTTTGCATC	Universal qPCR Primer*	57	
*dre-let-7d*	TGAGGTAGTTGGTTGTATGGTT	Universal qPCR Primer*	57	
*ago2.*	CACCACAAGAATATGTCTTCAAACCA	ACCATGTGCTCAACTATTTCACG	58	187
*Dicer*	TCAGGTTGAACTTCTTGAAGCAG	CTGAGCCACAGATGACGCT	58	175
*Drosha*	CTGAGAGACTTCGGCACCA	TCTCTGCTGCGGTGTCTC	58	199
*Zfmor*	CACCGAACGCACTTGCCATGA	GTGATGACATCTCCAGGACTAG	55	1194
*zfdor1*	ATGGAGCCGTCCGTCATTCCCG	CCCGTGGATCCTGTCCAGGCC	55	1144
*zfdor2*	ATGGAGCCTCCAACAGTGAC	TCATGTGGGCTGCTTGATTG	58	1122
*zfmor-3UTR*	GCAGGTATGACTAGTCCTGGAG	TACTTGGCAGTCTGCGAGAAC	60	1040

* oligo from NCodeTM miRNA First-Strand cDNA Synthesis and qRT-PCR Kits (Invitrogen).

Ta: annealing temperature.

In recent years, miRNAs acting post-transcriptionally have been shown to regulate mRNA expression. Thus, miRNAs binding to their mRNA targets induce a downregulation of gene expression via translational repression and/or mRNA degradation [15], [16]. Likewise, miRNAs can be regulated by many genes; among them some specific miRNAs could be regulated via MOR, where fentanyl downregulates miR-190 [17], [18] and morphine decreases miRNAs such as miR-28, miR-125b, miR-150, and miR-382 [19]. Moreover, miR-23b binding to the MOR 3'-untranslated region (3'UTR) suppresses MOR-protein production [20]. miRNAs regulate gene expression via translational repression, binding to mRNA 3'UTR [20]; binding to sites located within the amino acid coding sequence (CDS) of mRNA transcripts [21], and even to 5′UTR elements of mRNA [22]. Likewise, the lethal 7 (Let-7) family, the first miRNA identified in humans, is highly conserved across species in both sequence and function [23] and has been shown to be a modulator of many genes, among them MOR; binding in the CDS [21] and within 3′UTR elements of mRNA [23].

Studies in humans have revealed that prenatal cocaine exposure outcome in alterations of different organs (lungs, liver) and systems (the heart system [24]–[26] and the nervous system [27]–[30]). In spite of the efforts made to explain the mechanism of action of cocaine in the addiction process, no clear pathway has been found, that explains its effects. Hence, several animal models (rat, mouse, rabbits and chimpanzees) have been used trying to better understand the molecular mechanism of cocaine which could lead to a therapeutic solution of the problem of addiction. In this sense, zebrafish has emerged as a valuable vertebrate animal model for studying developmental processes and modeling human disease [31], [32], including the study of cocaine at the behavioural and molecular levels [33], [34], since this organism displays many benefits in comparison to other vertebrate animal models: small size, low cost, rapid development, the transparency of the embryos, ex vivo development, high fecundity, and transient genetic manipulation by microinjection of mRNA (overexpression) or antisense morpholino oligonucleotides (knockdown) [35].

Taking into account all of the above aspects, we used the zebrafish as a model organism to determine the effects produced by cocaine in the expression of opioid receptors, let-7d miRNA and to study the interrelationship between let-7d and opioid receptors along embryonic development.

In this investigation we report that the silencing and overexpression of opioid receptors altered the expression of miRNA *dre-let-7d* (in zebrafish) and their precursors, indicating a close relationship between them. Also, cocaine altered the expression of opioid receptors and *dre-let-7d,* which means that cocaine could be regulating the function of opioid receptors.

**Figure 1:**
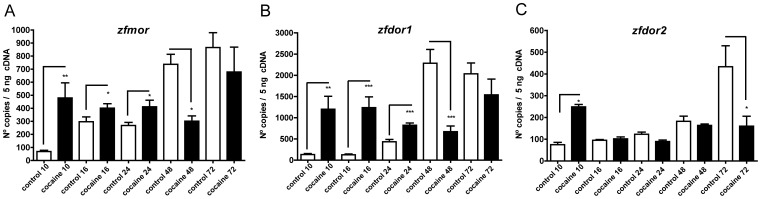
Effects of cocaine on the expression of *zfmor* (A), *zfdor1* (B) and *zfdor2* (C). qRT-PCR analyses of opioid receptor expression levels were studied at several development stages: 10, 16, 24, 48 and 72 hpf. Gene expression levels were measured as fluorescence intensities, using qPCR and were normalized to *ef1a* expression. Two hundred embryos were used to extract RNA and synthesize cDNA. Error bars represent means of mRNA copies at each developmental stage ± SEM. Three independent experiments (each one performed three times) were performed for each stage. *P*-Values were calculated with the two*-*tailed unpaired Student's t test: *P<0.05, **P<0.01, ***P<0.001.

**Figure 2:**
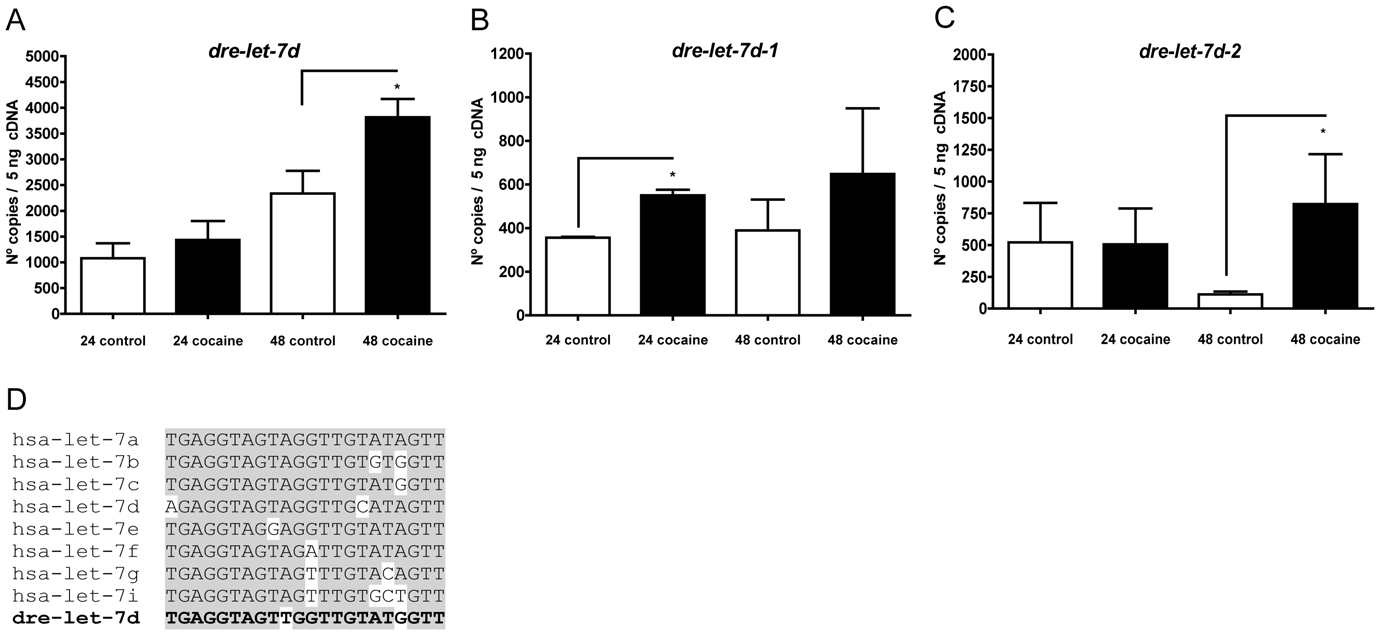
Effects of cocaine on the expression levels of *dre-let-7d* (A), *dre-let-7d-1* (B) and *dre-let-7d-2* (C). Total RNA was isolated from two hundred embryos and used to synthesize cDNA. Gene expression levels were measured as fluorescence intensities using qPCR and were normalized to *ef1a* expression. Two hundred embryos were used to extract RNA and synthesize cDNA. Error bars represent means of mRNA copies at each developmental stage ± SEM. Data are representative of three independent experiments and each experiment was performed three times. *P*-Values were calculated with the two*-*tailed unpaired Student's t test. D) DNA sequences of mature let-7 family members in vertebrates, humans and zebrafish. Sequence conservation is indicated by gray shading. The abbreviations of human (*Homo sapiens*) and zebrafish (*Danio rerio*) species is denoted as has and dre, respectively. The accession numbers (according to miRBase) of the several mature let-7 members were: hsa-let-7a (MI0000060), hsa-le-7b (MI0000063, hsa-let-7c(MI0000064, hsa-let-7d (MI0000065), hsa-let-7e (MI0000066), hsa-let-7f (MI0000067, hsa-let-7g (MI0000433), hsa-let-7i (MI0000434), dre-let-7d (MI0001868).

## Materials and Methods

### Animals

Adult zebrafish (*Danio rerio*, wild-type AB strain) were raised in a cycle of 14 h light: 10 h dark (LD) at 26°C in a multi-tank system at our Fish Facilities of the Institute of Neuroscience of Castile & Leon, belonging to the University of Salamanca. Embryos obtained from natural fertilization were selected using a Discovery.V8 stereomicroscope (Carl Zeiss, Germany), after which they were raised at 28.5°C and maintained in dishes containing sterile E3 medium (5 mM NaCl, 0.17 mM KCl, 0.33 mM CaCl, 0.33 mM MgSO4) in distilled water (Sigma, Madrid, Spain).

### Ethics Statement

All procedures and experimental protocols were carried out in accordance with the guidelines of the European Communities Council directive of 24 November 1986 (86/609/EEC), current Spanish Legislation (BOE 67/8509-12, 1998), and following the Guide for the Care and Use of Laboratory animals as adapted and promulgated by the US National Institute of Health. All efforts were made to minimize the number of embryos used and their suffering.

All experiments were performed at the University of Salamanca with the approval of the Animal Care Committee of this Institution.

**Figure 3:**
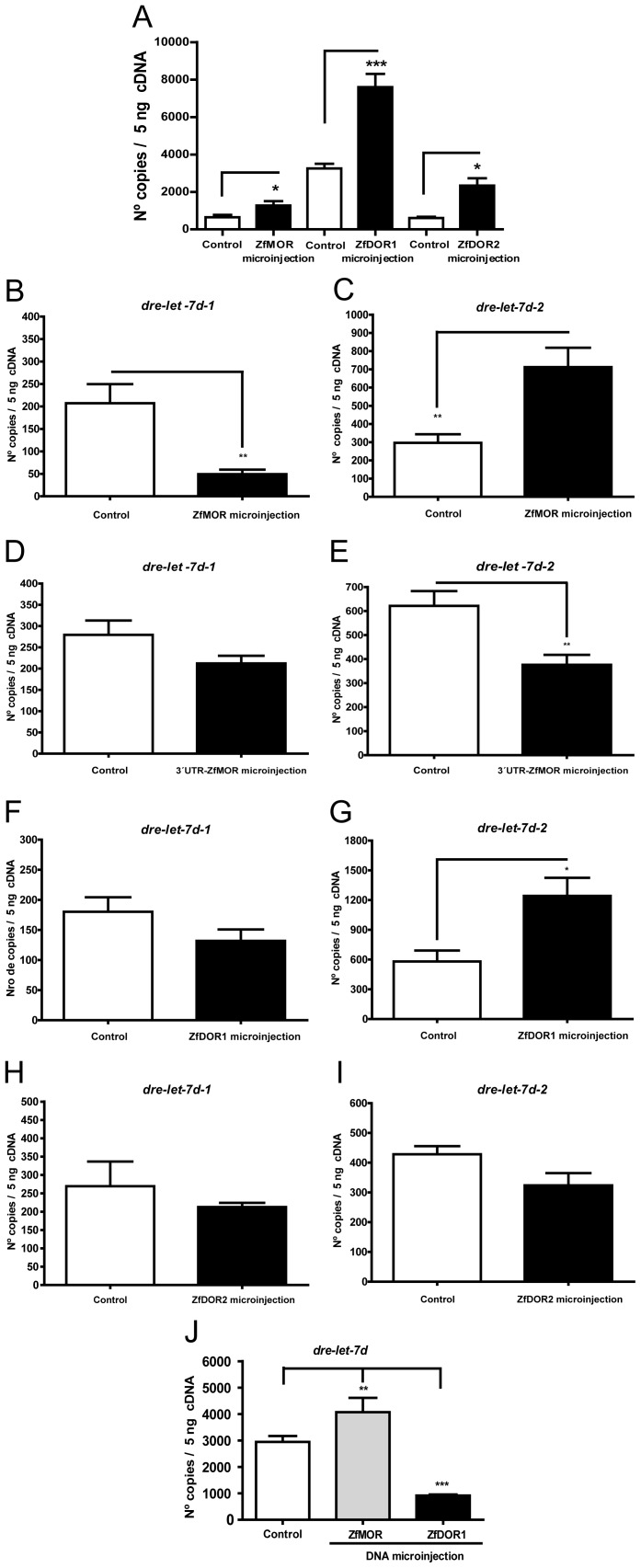
Expression of opioid receptors and *dre-let-7d* after opioid receptor DNA microinjections. (A) Quantification by qPCR of *zfmor*, *zfdor1* and *zfdor2* receptors at 48 hpf (after opioid receptor DNA microinjections). Expression of *dre-let-7d-1* (B, D, F and H), dre-let-7d-2 (C, E, G and I) and *dre-let-7d* (J) after opioid receptor DNA microinjections: 3'UTR-ZfMOR, ZfMOR, ZfDOR1 and ZfDOR2. Gene expression levels were measured as fluorescent intensities using qPCR and were normalized to *ef1a* expression. Two hundred embryos were used to extract RNA and synthesize cDNA. Error bars represent means of mRNA copies at each developmental stage ± SEM. Three independent experiments (each experiment performed three times) were performed for each stage. A-I; *P*-Values were calculated with the two-tailed unpaired Student's t test*:* *P<0.05, **P<0.01, ***P<0.001. J; data were analysed using ANOVA with the *post hoc* Dunnett test: *P<0.05, **P<0.01, ***P<0.001.

**Figure 4:**
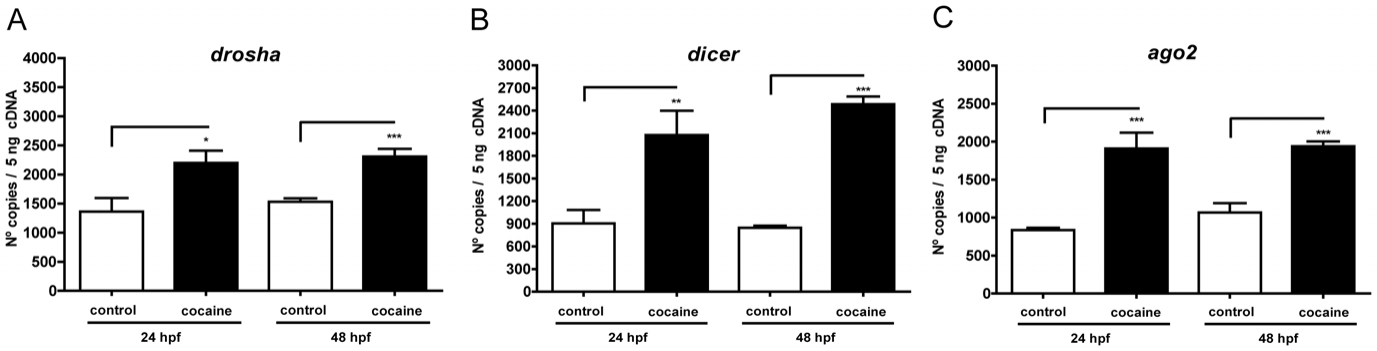
Effects of cocaine on the mRNA expression of proteins involved in the biogenesis of microRNAs. The expression levels of *drosha* (A), *dicer* (B) and *ago2* (C) were measured as fluorescence intensities using qPCR and were normalized to *ef1a* expression. Two hundred embryos were used to extract RNA and synthesize cDNA. Error bars represent means of mRNA copies at each developmental stage ± SEM. Three independent experiments (each experiment performed three times) were performed for each stage. *P*-Values were calculated with the two-tailed unpaired Student's t test: *P<0.05, **P<0.01, ***P<0.001.

### Drug Treatment

Cocaine HCl was dissolved in E3 medium and administered directly into Petri dishes containing the embryos. Thus, zebrafish embryos were exposed to 1.5 µM cocaine HCl at 5 hpf and when these embryos reached 8, 16, 24, 48 and 72 hpf of development they were harvested for RNA extraction and further cDNA synthesis. The dose of 1.5 µM cocaine HCl was chosen because this concentration is comparable to the plasma levels of benzoylecgonine, one of the main metabolites of cocaine, in human neonates [36]. Also, exposure to this concentration (with a non-anesthetic effect) and its later withdrawal causes anxiogenic effects in adult zebrafish [34].

### Determination of the Amount of Cocaine Entering Zebrafish Embryos

Embryos were exposed to 1.5 µM cocaine HCl from 5 to 24 hpf and from 5 to 48 hpf, and were then collected. Following this, embryos at both developmental stages were washed three times for 5 min each in E3 medium and dechorionated. Dechorionation was performed in order to quantify the real concentration of cocaine in the embryonic tissue, since the chorion might prevent cocaine from entering the embryo. Embryos were kept at 20°C. Samples were defrosted first at 4°C (1 h) and then at room temperature. After adding 1 ml of 10 mm ammonium formate, pH 9.3 [37], samples were homogenized mechanically with a Polytron device on ice. Homogenized embryonic tissue was centrifuged for 30 min at 4000 g at 4°C and the supernatants were collected and kept at 4°C [37] until high-performance liquid chromatography-mass spectrometry (HPLC-MS) analysis. Six samples per developmental stage (200 embryos were used per sample) were analysed by HPLC-MS. HPLC-MS analyses were performed as previously described [37] using a Waters ZQ 4000 with an Alliance HT HPLC apparatus. The HPLC conditions were as follows: column, Atlantis T3, 3 lm, 2.1 · 100 mm; solution A, 10 mm ammonium formate, pH 7.0, in H2O; solution B, methanol. Initial conditions were 30% B and a gradient was performed over 11 min to reach 100% B. The 286 and 289 u.m.a. signals were recorded and integrated in SIM mode. Cocaine-D was used as an internal deuterated standard (Cerrilliant, Round Rock, TX, USA).

**Figure 5:**
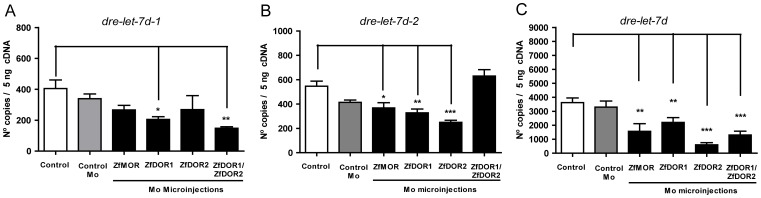
Effects of MOs on the expression levels of dre-let-7d and its precursors. Morpholinos of ZfMOR, ZfDOR1, ZfDOR2 ZfDOR1/ZfDOR2 were microinjected into the yolk in the one-to-four cell stage, and then the mRNA expression levels of *dre-let-7d-1* (A), *dre-let-7d-2* (B) and *dre-let-7d* (C) at 48 hpf were measured as fluorescence intensities using qPCR. One hundred and fifty embryos were used for RNA extraction. Each bar represents the number of copies of cDNA ± SEM. For each stage three experiments were performed, each performed three times. Between-group differences in *dre-let-7d* and its precursors were analysed using the ANOVA with the *post hoc* Dunnett test: *P<0.05, **P<0.01, ***P<0.001.

**Figure 6:**
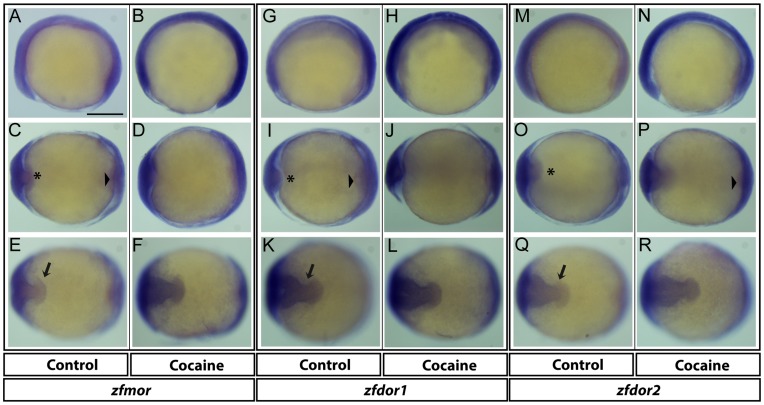
Expression of opioid receptors during the tail bud stage. Opioid receptors: *zfmor* (A, C and E), *zfdor1* (G, I and K) an*d zfdor2* (M, O and Q) were expressed during the tail bud stage (approximately 10 hpf). In all panels, the anterior aspect is to the left. For each opioid receptor, the left and right sides represent the embryos untreated and treated with cocaine respectively. A and B, lateral views (applies to G and H, M and N). C-F, animal pole views (applies to I-L, O-R). Arrowhead points to the future region of the tail bud, arrow indicates CNS development. Asterisks show the polster. Scale bar: 250 µm in A (applies to B-R).

### RNA Extraction and qRT-PCR

Total RNA, including miRNA, was extracted using Trizol® reagent (Invitrogen Corp. Carlsbad, CA, USA) following the protocol recommend by the manufacturers. In all cases, RNA samples were treated with DNase I (Roche Scientific, Madrid, Spain) following the protocol recommended by the manufacturers. RNA concentrations were determined using a NanoDrop (NANODROP 2000C, Thermo Scientific) spectrometer. Samples were assayed three times and mean values were recorded. cDNA synthesis was carried out by reverse transcription of total RNA to cDNA using the Promega Corp. (Madison, WI, USA) reverse transcription kit. The RNA was combined with dT oligonucleotide in a total volume of 5 µl and incubated at 70°C for 5 min. The final volume of each reaction was brought up to 20 µl by the addition of 4 µl of retrotranscriptase, 3 µl MgCl and 5.5 µl diethyl pyrocarbonate-treated water. Samples were then incubated for 10 min at 25°C, followed by 1 h at 42°C and 15 min at 70°C. The cDNA samples were then treated with RNase A (20 ng/µl for 7–8 µg cDNA) for 20 min at 37°C. Following this, these products were purified using the QIAquick PCR Purification Kit (QIAGEN) and eluted in DNase-free water. The quantity and quality of the cDNA was determined by measuring absorbance at 260 nm and 260 nm/280 nm with NanoDrop technology. PCR products were measured using the curve of the SYBR-Green method. SYBR-Green was included in a 2x Master Mix from Applied Biosystems (Alcobendas, Madrid, Spain) (SYBR Green dye, dNTPs, Passive Reference (ROX), Amplitaq1 Gold DNA polymerase). The ABI Prism 7300 detection system (Applied Biosystems, Madrid, Spain) was used to amplify the different genes. The oligonucleotides used to amplify the different genes and the annealing temperatures are shown in table 1. Between three and six samples were taken for each gene to perform the PCR reactions, and the experiments were repeated three times for each gene. The final volume of each reaction was 20 µl: 10 µl of Master Mix, 1 µl of each oligonucleotide, 7 µl of distilled water and 1 µl of cDNA at a concentration of 5 ng/µl. *ef1α* was used as a reference gene.

**Figure 7:**
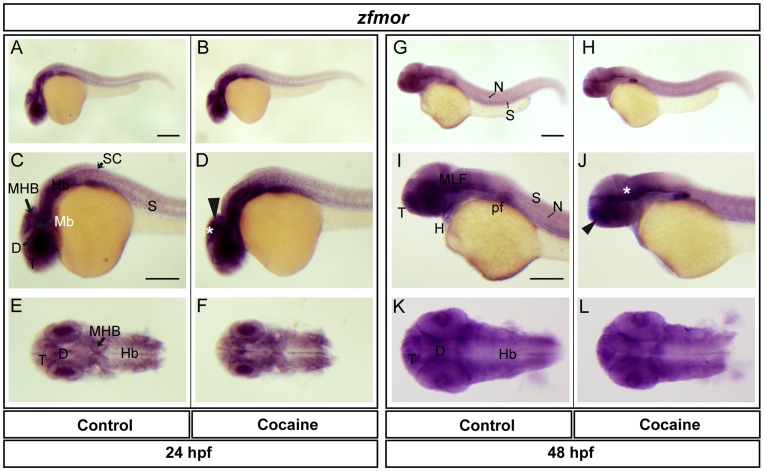
Spatial distribution of *zfmor* at 24 and 48 hpf. Lateral views (A-D, G-J), dorsal views (E-F, K-L). *zfmor* was found in the telencephalon, diencephalon, midbrain (optic tectum), MHB, hindbrain, spinal cord, eye and somites. The expression in embryos exposed to cocaine exposure was increased in the optic tectum and MHB (Asterisk and arrow, respectively). At 48 hpf, *zfmor* was expressed to a similar extent to what was observed at 24 hpf, and also in the MLF, notochord, pectoral flipper and heart. Cocaine induced a decrease in *zfmor* levels in the telencephalon and MLF (arrow and asterisk, respectively). Abbreviations: T: telencephalon; Mb: midbrain, Hb: hindbrain, D: diencephalon, N: notochord; S: somite; pf: pectoral flipper; H: heart. Scale bar: 200 µm in A (Applies to B, G and H) and 250 µm in C (Applies to D, E, F, I, J, K and L).

**Figure 8:**
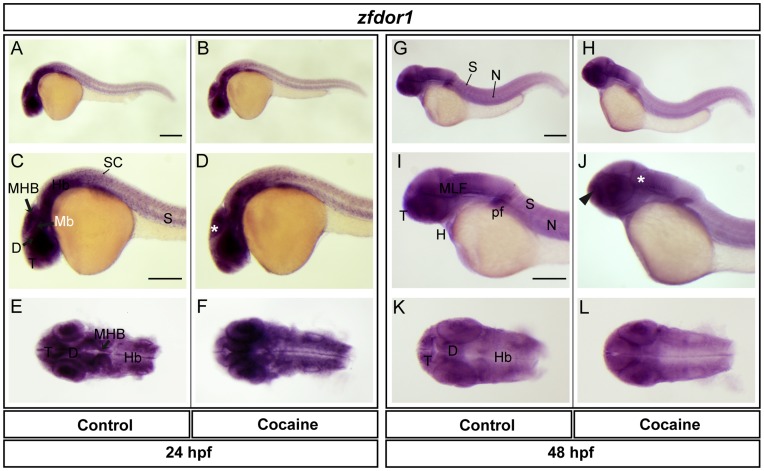
Spatial distribution of *zfdor1 a*t 24 and 48 hpf. Lateral views (A-D, G-J), dorsal views (E-F, K-L). *zfdor1* was found in telencephalon, diencephalon, midbrain (optic tectum), MHB, hindbrain, spinal cord, eye and somites. Expression in embryos exposed to cocaine in the optic tectum (asterisk), telencephalon, midbrain, hindbrain and spinal. At 48 hpf *zfdor1* was expressed to a similar extent as at 24 hpf, but also with expression in the MLF, notochord, pectoral flipper and heart. Cocaine induced a decrease in expression in the telencephalon and MLF (arrowhead and asterisk, respectively). Abbreviations: T: telencephalon; Mb: midbrain, Hb: hindbrain, D: diencephalon, N: notochord; SC: spinal cord; S: somite; pf: pectoral flipper; H: heart. Scale bar: 200 µm in A (Applies to B, G and H) and 250 µm in C (Applies to D, E, F, I, J, K and L).

### 3.6. Standard Curves

A basic PCR was carried out to amplify the fragment of the transcripts of interest (*zfmor, zfdor1, zfdor2, dre-let-7d, dre-let-7d-1, dre-let-7d-2* and *ef1α*) in zebrafish cDNA with the same pair of primers as those to be used in the real-time PCR (qPCR). The PCR products were visualized on agarose gel and the fragment corresponding to each gene was cut from agarose gel and then purified. Serial 1∶10 dilutions were made from the purified PCR product, ranging from 10^−2^ ng/µl to 10^−5^ ng/µl and chosen according to their cycle quantification (Cq) values and considering that perfect amplification had been obtained when the difference in the Cq values from one dilution to the next was close to 3.3, which afforded a four-point straight line with a slope of −3.3. The numbers of cDNA copies were calculated using the following formula:

1. Number of bases of the amplicon×330 Da/base×2 bases/base pair (the purified product is in double-stranded DNA) = X g/mol (weight of the amplicon).
2. Weight of the amplicon/6.023×10^23^ = Y g/amplicon molecule.
3. Dilution concentration/Y g/amplicon molecule = Z molecules/µl.

### ZfMOR, ZfDOR1, ZfDOR2 and ZfMOR 3`UTR Cloning and Microinjection

The complete sequences of ZfMOR, ZfDOR1, ZfDOR2 and ZfMOR 3′UTR were amplified by PCR using specific primers (shown in table 1) using the TaKaRa LA Taq™ kit (Takara Bio Inc.). The program used for amplification was as follows: 5 min at 95°C followed by 35 cycles of 15 s at 95°C, 30 s at 55–60°C, and 1 min at 70°C. At the end of the cycles, a final extension temperature of 70°C was added for 10 min. Then, the PCR products were purified and cloned into the pCR^®^II vector (Invitrogen). TOP 10′F cells (Invitrogen) were transformed with the constructs, and miniprep (ZYMO Research Corporation, CA, USA) and midiprep (Sigma, St. Louis, USA) were performed to obtain high quantities of the constructs, which were sent for sequencing. Then, the products obtained after digestion with EcoRI for 1 h at 37°C, were purified for the injection of the DNA (500 pg/3 nl) into one-cell zebrafish embryos using a micromanipulator-microinjector system from Eppendorf AG (Hamburg, Germany).

**Figure 9:**
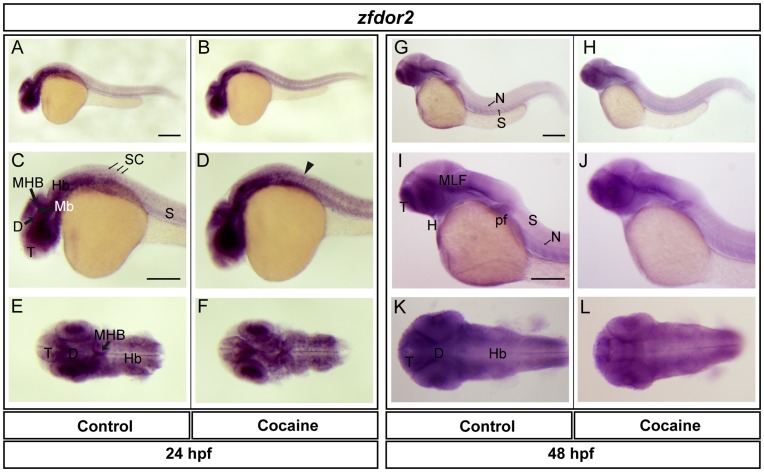
Spatial distribution of *zfdor2 a*t 24 and 48 hpf. Lateral views (A-D, G-J), dorsal views (E-F, K-L). At 24 hpf, *zfdor2* was found in the telencephalon, diencephalon, midbrain (optic tectum), MHB, hindbrain, spinal cord, eye and somites. At 48 hpf, *zfdor2* was expressed to a similar extent as at 24 hpf, also with expression in the MLF, notochord, pectoral flipper and heart. Cocaine did not induce evident changes in the expression of *zfdor2* at 24 hpf (except in the spinal cord, where its expression was increased (arrowhead), or at 48 hpf. Abbreviations: T: telencephalon; Mb: midbrain, Hb: hindbrain, D: diencephalon, N: notochord; SC: spinal cord; S: somite; pf: pectoral flipper; H: heart. Scale bar: 200 µm in A (Applies to B, G and H) and 250 µm in C (Applies to D, E, F, I, J, K and L).

### Morpholino Microinjection

Morpholino antisense (MOs) oligomers were used for opioid receptor knockdown (Gene Tools, LLC Philomath, OR) and to study the expression of *dre-let-7d* and its precursors (*dre-let-7d-1* and *dre-let-7d-2*). The sequences used to knockdown the opioid receptors were ZfMOR (AATGTTGCCAGTGTTTTCCATCATG), ZfDOR1 (GAATGACGGACGGCTCCATCGCTTC), and ZfDOR2 (GGAGGCTCCATTATGCTCGTCCCCT).

These MOs were diluted (in sterilized water) to a stock concentration of 0.3 mM and the concentrations employed for the different opioid receptors were 0.2 µM (ZfMOR), 1 µM (ZfDOR1), 1 µM (ZfDOR2) and 1 µM (ZfDOR1-ZfDOR2). The MO experimental groups were ZfMOR, ZfDOR1, ZfDOR2 and ZfDOR1-ZfDOR2 and the MO control group (microinjected with a standard MO control solution (CCTCTTACCTCAGTTACAATTTATA)). Approximately, 3nl of each MO was microinjected into the yolks of zebrafish embryos at the one-to-four-cell stage according to published protocols [38] with a micromanipulator-microinjector system from Eppendorf AG (Hamburg, Germany).

### Whole-mount in Situ Hybridization (*ISH*)

Embryos at 24 hpf and 48 hpf were dechorionated, fixed with 4% paraformaldehyde (PFA) in phosphate saline buffer (PBS) overnight at 4°C, washed twice in PBS, 5 min each at room temperature (RT), and finally maintained in absolute methanol at −20°C until use. Embryos at 10 hpf first were fixed with PFA and then dechorionated. The following day, the embryos were rehydrated in consecutive dilutions of methanol/PBS (75, 50 and 25%), 5 min for each dilution. These were washed 4 times for 5 min each in 100% PBS-Tween 20 (0.2%) (PBT). Proteinase K (10µg/ml) was used to permeabilize the embryos at RT over 20 min for 24 hpf embryos and 40 min for 48 hpf embryos (Embryos at 10 hpf were not permeabilized). Proteinase K digestion was stopped by incubating the embryos for 20 minutes in 4% (wt/vol) (PFA) in 1X PBS. Four washes, 5 min per wash, in 1X PBT were performed to remove residual PFA. After 2h of prehybridization (with ZfMOR, ZfDOR1, and ZfDOR2 riboprobes, each one separately) the embryos were left overnight at 64°C to hybridize. Washes were performed every 20 min in each wash solution with prehybridization/Tris-buffered saline (TBS) (50%/50%) and TBS-Tween 20 (0.2%) (TBST) over 2 hours. Then, the embryos were blocked with blocking buffer (goat serum+TBST) for 2 hours and were incubated overnight with antidigoxigenin antibody conjugated with alkaline phosphatase (1∶3000, Roche) at 4°C. The following day, the embryos were washed with Xpho solution (1M Tris HCl, pH 9.5, 1M MgCl_2_, 4M NaCl and 20% Tween-20) for 10 minutes over 1 hour, and finally the hybridization was developed with a fresh NBT/BCIP mix (Roche). The dre-let-7d miRNA riboprobe was purchased from Exiqon, and the *ISH* experiment was performed according to the manufacturer’s protocol (miRCURY LNA™ microRNA Detection Probes for *in situ* hybridization, Exiqon).

### Immunohistochemistry

Cell proliferation was analysed using phosphorylated histone-3 (H3P) as a cellular division marker [39] and p53 tumour suppressor as an inducer of cell apoptosis [40]. Embryos at 24 and 48 hpf were fixed in 4% (w/v) PFA (Sigma) overnight at 4°C and stored in methanol (Panreac) at −20°C. Following this, they were rehydrated two times in 0.2% PBS for 5 min and then blocked in PBT-20 with 10% (v/v) goat serum (Sigma) and 1% BSA (10X) for 4h at RT. For immunodetection, embryos were incubated with a mouse anti-H3P antibody (Ab) (1: 500; Abcam, Cambridge, UK) and a rabbit anti-p53 Ab (5∶500) overnight at RT. Then, the embryos were washed in PBS and incubated for 1 h at RT in PBST and 1% BSA (10X) with secondary Abs goat Alexa-546 anti-mouse and goat Alexa-488 anti-rabbit (1∶500; Invitrogen, Barcelona, Spain). After incubation with these Abs, the embryos were washed again with PBS.

**Figure 10:**
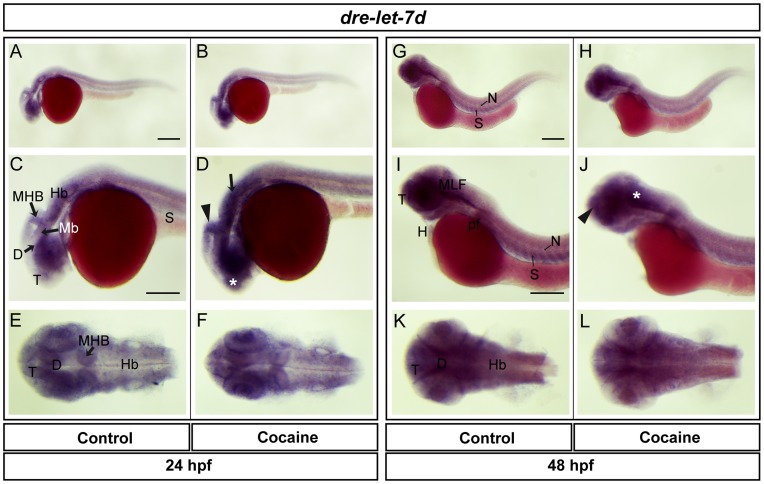
Spatial distribution of *dre-let-7d a*t 24 and 48 hpf. Lateral views (A-D, G-J), dorsal views (E-F, K-L). *dre-let-7d* was found in the telencephalon, diencephalon, midbrain (optic tectum), MHB, hindbrain, spinal cord, eye and somites. Expression in embryos exposed to cocaine was observed in the telencephalon, MHB, and hindbrain (asterisk, arrowhead and arrow, respectively). At 48 hpf, *dre-let-7d* was expressed to a similar extent to the situation at 24 hpf, but also with expression in the MLF, notochord, pectoral flipper and heart. Cocaine induced an increase in expression in the telencephalon and MLF (arrowhead and asterisk, respectively). Abbreviations: T: telencephalon; Mb: midbrain, Hb: hindbrain, D: diencephalon, N: notochord; S: somite; pf: pectoral flipper; H: heart. Scale bar: 200 µm in A (Applies to B, G and H) and 250 µm in C (Applies to D, E, F, I, J, K and L).

**Figure 11:**
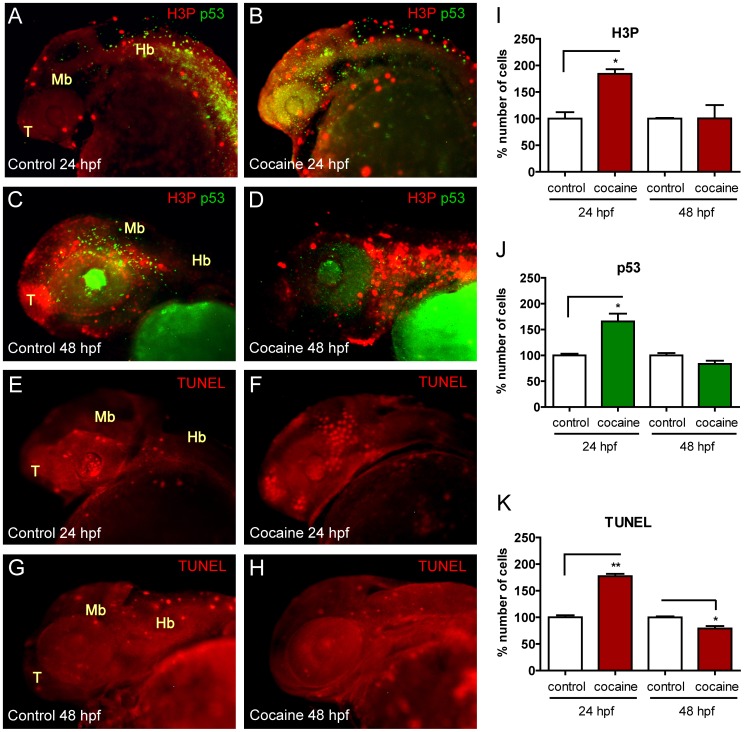
Analyses of proliferation and apoptosis in whole-mount embryos by IHC and TUNEL assays. Lateral views of embryos not exposed to cocaine are on the left side (A, C, E and G) and those exposed to the drug are on the right (B, D, F and H). Cocaine-exposed embryos showed an increase in apoptotic cells (p53) at 24 hpf (A vs. B) and a decrease at 48 hpf (C vs. D). This finding was similar with the TUNEL assay method at 24 hpf (E vs. F) and 48 hpf (G vs. H). The number of proliferating cells (in percent) (H3P) is increased both at 24 and 48 hpf (A vs. B and C vs. D, respectively). I, J and K show the percent changes in H3P, p53, and TUNEL at 24 and 48 hpf. Abbreviations: H3P: phosphorylated histone-3; p53: p53 protein; TUNEL: Terminal deoxynucleotidyl transferase dUTP nick end labeling; T: telencephalon; Mb: midbrain; Hb: hindbrain. Scale bars 150 µm. *P*-Values were calculated by *two*-*tail unpaired student's t test:* *P<0.05, **P<0.01.

### Apoptosis Assays

To determine apoptosis, we used *the In Situ Cell Death Detection Kit, AP* (Roche) following the manufacturer’s protocol (with slight modifications made by our group) for whole-mount zebrafish embryos. The embryos were fixed overnight with 4% PFA and stored in absolute methanol at −20°C, after which they were rehydrated by successive dilutions of methanol in PBS (75% methanol, 50% methanol, 25% methanol) and washed 4 times with PBST. Embryos were further permeabilized with PK (10 µg/ml) at RT for 20 min (24 hpf) and 40 min (48 hpf). Following this postfixation with PFA, embryos were postpermeabilized by incubation with 0.1% Triton X-100 in 0.1% (freshly prepared) sodium citrate for 1h. They were then incubated with the TUNEL reaction mix (enzyme and label solution mixed (1∶5)) for 1h at 37°C in the dark. Next, the embryos were washed three times with PBS (5 min per wash), after which the samples were incubated in Converter-AP (alkaline phosphate) for 30 min at 37°C, followed by three washes with PBS (5 min per wash). Finally, apoptosis was detected using Fast Red tablets (Roche) (AP substrate).

### Image Analysis

The images obtained with whole-mount IHC and *ISH* were documented with a fluorescence stereoscope (Leica M165 FC) using 6.3x and 12x magnifications and a numerical aperture of 0.30. All images were processed with Photoshop CS5 software (Adobe System Inc.). The numbers of proliferating and apoptotic cells were counted with ImageJ software (NCBI).

**Figure 12:**
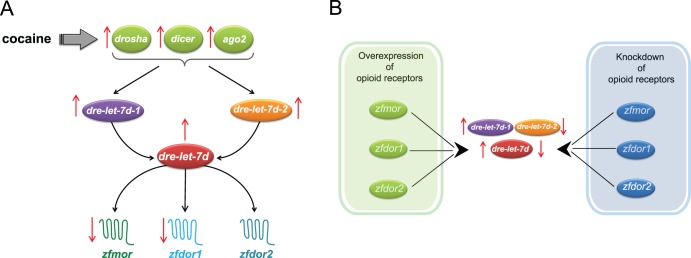
Schematic process showing the hypothetical cocaine actions in zebrafish embryos at 48 hpf. It is proposed that cocaine down-regulate opioid receptors via miRNA *dre-let-7d*. A) Cocaine altering the expression of the protein of miRNA biogenesis (*drosha, dicer* and *ago2*) affect the expression of the miRNA *dre-let- 7d* and its precursors (*dre-let-7d-1* and *dre-let-7d-2)*. Hence, as consequence, a downregulation of opioid receptors, *zfmor, zfdor1 and zfdor2,* is induced. B) Overexpression (by DNA microinjections) and knockdowns (by MO microinjections) of opioid receptors, ZfMOR, ZfDOR1 and ZfDOR2, affect the expression of *dre-let-7d* and its precursors (*dre-let-7d-1* and *dre-let-7d-2*), indicating a close interrelationship between opioid receptors and miRNA let-7d.

### Statistical Analyses

The real-time PCR results are represented as means ± SEM and were analysed using the two-tailed Students t-test between the treatment group and the control group. One-way analysis of variance (ANOVA) followed by Tukey post-hoc and Dunnet test comparison were used to compare gene expression between the different treatment groups and the control group. In all tests, the difference was considered significant if *P*<0.05. Statistical analyses were performed with Prism 5 (GraphPad Software Inc.).

## Results

### The Amount of Cocaine Absorbed by the Zebrafish Embryos

Using the HPLC-MS analysis, we observed that 0.175±0.0472 nm of cocaine was detected in each zebrafish embryo exposed to 1.5 µM cocaine-HCl from 5 to 24 hpf and from 5 to 48 hpf. The value of the amount of cocaine represents approximately 12% of the initial concentration of 1.5 µM cocaine used.

### Cocaine Affects the Expression Levels of Opioid Receptors

To address whether cocaine affects the expression levels of ZfMOR [41] (GenBank accession No.: NM_131707) and of the delta opioid receptors duplicates, ZfDOR1 [42], [43] (GenBank accession No.: NC_007130.2) and ZfDOR2 [44] (GenBank accession No.: NM_212755), we studied zebrafish embryos during several embryonic developmental stages: 10, 16, 24, 48 and 72 hpf. Embryos exposed to cocaine HCl showed different responses, depending on the opioid receptor and the developmental stage. The expression of *zfmor* assessed by qPCR was upregulated (Fig. 1A) at 10 hpf, 16 hpf and 24 hpf with respect to the control group (*P* = 0.001, *P* = 0.04 and P = 0.04, respectively), while at 48 hpf *zfmor* was downregulated (*P* = 0.001). At 72 hpf, no substantial changes were observed. Similar to the case of *zfmor*, cocaine produced an increase in *zfdor1* (Fig. 1B) at 10 hpf, 16 hpf and 24 hpf with respect to the control groups (*P* = 0.001, *P* = 0.0004 and *P* = 0.001, respectively), while at 48 hpf *zfdor1* was downregulated (*P* = 0.001). At 72 hpf cocaine did not induce important changes in *zfdor1* expression. Also, cocaine increased the expression of *zfdor2* only at 10 hpf (*P* = 0.001) (Fig. 1C) and decreased it at 72 hpf (*P* = 0.015). The duplicated *zfdor2* showed less change after exposure to cocaine in the other developmental stages.

### Cocaine Modulates the Expression of *dre-let-7d* and its Precursors (RT-qPCR study)

The human miRNA *let-7d* showed high homology with zebrafish *dre-let-7d* (72%), and *dre-let-7d* displayed high homology (from 63 to 81%) with the other family members of human let-7 (let-7a, let-7b, let-7c, let-7e, let-7f, let-7g and let-7i) (Fig. 2D). To address whether cocaine affected the expression of *dre-let-7d* (MIMAT0001762) and its precursors, *dre-let-7d-1* (MI0001868) and *dre-let-7d-2* (MI0001870) (the sequences were obtained from miRBase: http://www.mirbase.org), embryos were exposed to 1.5 µM cocaine HCl at 5 hpf and when they reached 24 and 48 hpf of development the embryos were sacrificed. The expression profile, determined by qPCR, revealed a higher expression of *dre-let-7d* at 48 hpf with respect to 24 hpf. Moreover, embryos exposed to cocaine HCl displayed an upregulation of *dre-let-7d* expression levels at 24 and 48 hpf (Fig. 2A) with respect to their control groups; the difference was only statistically significant at 48 hpf (*P* = 0.003). The precursor *dre-let-7d-2* had a higher expression than *dre-let-7d-1* at 24 hpf, whereas at 48 hpf *dre-let-7d-1* was expressed almost three times more with respect to *dre-let 7d-2*. When embryos were exposed to cocaine HCl they showed an increase in *dre-let-7d-1* (Fig. 2B) at 24 hpf and 48 hpf. A significant difference was only found at 24 hpf (*P* = 0.035). Additionally, after cocaine exposure *dre-let-7d-2* only showed an increase in the level of transcription at 48 hpf (Fig. 2C) (*P* = 0.04), with no substantial changes at 24 hpf.

### Opioid Receptor DNA-microinjection Modulates the Expression of *dre-let-7d* and its Precursors (qPCR studies)

The human let-7 miRNA family has a putative binding site at the µ-opioid receptor (let-7d in the CDC) [21] and within the 3′UTR elements of mRNA MOR [23]. To address whether the dre-let-7d miRNA precursors (*dre-let-7d-1* and *dre-let-7d-2*) and the mature *dre-let-7d* were endogenous regulators of opioid receptors, we microinjected the DNA sequences of opioid receptors, (ZfMOR, ZfDOR1 and ZfDOR2) and evaluated the expression of *dre-let-7d* and its precursors. DNA microinjection of opioid receptors (ZfMOR, ZfDOR1 and ZfDOR2) (overexpression) at the single-cell stage induced an upregulation of the expression of *zfmor, zfdor1 and zfdor2* at 48 hpf (Fig. 3A) (*P* = 0.039, *P* = 0.0002 and *P* = 0.023, respectively). *zfmor* showed a higher transcript expression with respect to both *zfdor1* and *zfdor2*. These findings show that the overexpression of opioid receptors would be due to microinjection of their respective DNA sequences.

When the ZfMOR DNA sequence was injected, *dre-let-7d-1* (Fig. 3B) decreased and *dre-let-7d-2* (Fig. 3C) increased the amount of mRNA at 48 hpf; in both cases, the changes were statistically significant. The microinjection of ZfMOR 3′UTR DNA did not change the expression of *dre-let-7d-1* (Fig. 3D). Nevertheless, following ZfMOR 3′UTR DNA injection the expression of *dre-let-7d-2* was decreased (Fig. 3E, *P* = 0.002) at 48 hpf. ZfDOR1 DNA microinjection into zebrafish embryos did not alter the transcription of *dre-let-7d1* (Fig. 3F), while *dre-let-7d-2* was upregulated (Fig. 3G, *P* = 0.02). When ZfDOR2 DNA was microinjected at the single-cell stage, embryos did not display substantial changes in the expression of *dre-let-7d-1* (Fig. 3H) and *dre-let-7d-2* (Fig. 3I). Finally, ZfMOR DNA microinjection at the single-cell stage and evaluated a 48 hpf upregulated the mRNA amount of *dre-let-7d* (Fig. 3J) while ZfDOR2 DNA injection downregulated *dre-let-7d* (Fig. 3J).

### Cocaine Affects the Proteins Involved in miRNA Biogenesis

miRNA biogenesis starts with pri-miRNA transcription, after which Drosha processes the pri-miRNA and produces the pre-miRNA hairpin precursor. Dicer, in the cytoplasm, cleaves the pre-miRNA into a mature miRNA [45]. Argonaut proteins are the catalytic components of RISC and of all the Ago proteins, only Ago2 displays endonuclease activity [15]. In light of the above, the changes produced by cocaine in these proteins could have consequences in the expression of miRNA precursors. Our qPCR experiments showed that embryos exposed to 1.5 µM cocaine HCl at 5hpf showed an increase in Drosha, Dicer and Ago2 at both 24 and 48 hpf. Drosha mRNA was upregulated (from 1362 to 2198 mRNA copies) at 24 hpf (*P = *0.02) and at 48 hpf (from 1530 to 2309 copies) (*P* = 0.0001) (Fig. 4A). Dicer mRNA underwent an upregulation at 24 hpf (from 904 to 2076 copies) (*P* = 0.002) and 48 hpf (from 845 to 2485 copies) (*P* = 0.001) (Fig. 4B). Finally, the expression of Ago2 mRNA was increased at 24 (from 835 to 1098 copies) and 48 hpf (from 1066 to 1939 copies) (Fig. 4C), in both cases being statistically significant (*P* = 0.0001 and *P* = 0.0002), respectively.

### Opioid Receptor Knockdown Affects the Expression of *dre-let-7d* and its Precursors (qPCR studies)

Since opioid receptors (MOR) are considered putative targets of the let-7 miRNA family [21], [23], we speculated that silencing the opioid receptors would affect the expression of *dre-let-7d* and its precursors. MO microinjections of opioid receptors did not alter the morphology of embryos, but a delay in the development of zebrafish embryos was observed with regard to the control group (embryos without microinjection of MOs). The absence of ZfMOR, ZfDOR1, ZfDOR2 and ZFDPOR1/2, due to MO microinjection into the yolk at the one-to-four-cell stage, affected the expression of *dre-let-7d* and its precursors at 48 hpf. When ZfDOR1 and ZfDOR1-ZfDOR2 MOs were microinjected, we found a decrease in *dre-let-7d-1* (Fig. 5A, *P* = 0.05). ZfMOR, ZfDOR1, ZfDOR2 MOs microinjection induced a decrease in *dre-let-7d-2* (Fig. 5B, *P* = 0.05). Additionally, MOs microinjections of ZfMOR, ZfDOR1, ZfDOR2 and ZfDOR1-ZfDOR2 downregulated the expression profile of the mature dre-let-7d miRNA (Fig. 5C, *P* = 0.05). To establish that the observed changes due to MO microinjections were due to action of MOs on their targets, we employed two control groups: embryos that were injected with standard MO (not targeting any gene in zebrafish, named MO control group) and embryos without MO injection (the control group). The standard MO control group permitted us to distinguish whether changes in mRNA expression levels were due to the actions of MOs or were caused by the mechanical alteration produced by the microinjection needle or were due to the pressure of the volume deriving from cell microinjection.

### Effects of Cocaine on the Expression Pattern of Opioid Receptors

The high expression of opioid receptors measured by qPCR during the early stage of zebrafish embryonic development (10 hpf) (Fig. 1A, B and C) attracted our attention, since the presence of opioid receptors in the early developmental stage means that opioid receptors play an important role in embryogenesis. Thus, with our qPCR data we studied the spatial distribution of opioid receptors by *ISH*. This is the first time that opioid receptors have been shown to be expressed (using riboprobes) during the late process of gastrulation in zebrafish (approximately 10 hpf). *zfmor* was expressed in the neural plate, in the future cephalic structures along the entire embryonic axis, the posterior trunk and the tail bud region (Fig. 6A, C and E). Additionally, *zfmor* was detected in the anterior region of the precordal plate (vegetal-pole view). A dorsal view (Fig. 6E) revealed that *zfmor* was expressed at the site of the future cephalic structures of the neural plate and was also expressed in the future tail region. Exposure to cocaine at 5 hpf induced an increase in the expression of *zfmor* in the future cephalic structures along the entire embryonic axis, posterior trunk and the tail bud regions (Fig. 6B, D and F). The spatial distributions of *zfdor1* (Fig. 6G, I and K) and *zfdor2* (Fig. 6M, O and Q) were found in regions similar to those observed for *zfmor* (in the future cephalic structures along the entire embryonic axis, posterior trunk and tail bud region). Cocaine-exposed embryos showed an unpregulation of *zfdor1* (Fig. 6H, J and L) and *zfdor2* (Fig. 6N, P and R) in these regions.

We also studied the expression of ZfMOR, ZfDOR1 and ZfDOR2 (at 24 and 48 hpf, using their respective riboprobes. The z*fmor* transcript was found in the telencephalon, diencephalon, optic tectum, midbrain, midbrain-hindbrain boundary (MHB), hindbrain, spinal cord, eye and somites (Fig. 7A, C and E). In cocaine-exposed embryos the expression of *zfmor* in the optic tectum and MHB (Fig. 7B, D and F) was increased. At 48 hpf, the distribution of *zfmor* was very similar to that observed at 24 hpf, and it was also expressed in the medial longitudinal fascicle (MLF), notochord, pectoral flipper and heart (Fig. 7G, I and K). Cocaine induced a decrease in the expression of *zfmor* in the telencephalon and MLF and hindbrain (Fig. 7H, J and L) at 48 hpf. In the case of *zfdor1,* it was distributed in the same areas as *zfmor* at 24 hpf: namely, the telencephalon, diencephalon, midbrain (optic tectum), MHB, hindbrain, spinal cord, eye and somites (Fig. 8A, C and E). At 48 hpf *zfdor1* was expressed in the same regions as at 24 hpf and also in the MLF, notochord, heart, pectoral flipper and somites (Fig. 8G, I and K). Cocaine induced a slight upregulation of *zfdor1* in the CNS at 24 hpf (telencephalon, midbrain and hindbrain) (Fig. 8B, D and F), while at 48 hpf the expression of *zfdor1* was decreased in the telencephalon and MLF (Fig. 8H, J and L). In the case of *zfdor2* at 24 hpf, like *zfmor* and *zfdor1* this receptor was expressed in the telencephalon, diencephalon, midbrain (optic tectum), MHB, hindbrain, eye and somites (Fig. 9A, C and E), while at 48 hpf it was expressed in the same areas and also in the MLF, notochord, pectoral flipper and heart (Fig. 9G, I and K). Exposure of embryos to cocaine HCl (at 5hpf) did not elicit evident changes in the pattern of expression of *zfdor2* at 24 hpf (except in the spinal cord, where its expression was increased (Fig. 9B and D) or at 48 hpf (9H, J and L).

### Effects of Cocaine on the Spatial Expression of *dre-let-7d*

Using LNA™ Probes we found *dre-let-7d* to be distributed in the telencephalon, diencephalon, midbrain (optic tectum), hindbrain, eye and somites (Fig. 10A, C and E) at 24 hpf. At 48 hpf, *dre-let-7d* was distributed in the same regions as at 24 hpf and also in the MLF, notochord, pectoral flipper and heart (Fig. 10G, I and K). According to *ISH*, the expression of *dre-let-7d* was increased in embryos exposed to cocaine at 24 hpf in the telencephalon, midbrain, MHB and hindbrain (Fig. 10B, D and F), and at 48 hpf cocaine upregulated the expression of *dre-let-7d* in the telencephalon and MFL (Fig. 10H, J and L). It is important to note that cocaine exposure produced an increase in the expression of *dre-let-7d* as observed by *ISH* (Fig. 2A) at 48 hpf. Surprisingly, this result is opposite to the expression of *zfmor* and *zfdor1,* where a decrease was observed also at 48 hpf.

### Cocaine Produces Changes in the Processes of Proliferation and Apoptosis

Our IHC studies revealed that cocaine increased the number of proliferating cells (Fig. 11A and B) at 24 hpf in 80% of H3P (H3P-measured in percent) (Fig. 11I) (*P* = 0.030). Likewise, at 48 hpf cocaine induced an increase in H3P (Fig. 11C and D) of 5% with respect to the control group (Fig. 11I). In the case of apoptotic cells, embryos exposed to cocaine (p53-measured in percent) had 65%-increased levels of p53 (Fig. 11A and B) at 24 hpf (*P* = 0.0495) (Fig. 11J). At 48 hpf, cocaine decreased p53 levels (Fig. 11C and D) by 17% (Fig. 11I). Finally, with the TUNEL assay we found that the number of apoptotic cells (measured in percent %) was increased (by cocaine exposure at 5hpf) at 24 hpf (Fig. 11E vs. F) in 77% (*P* = 0.0012) (Fig. 11K) and decreased at 48 hpf (Fig. 11G vs. H) in 22% (*P* = 0.0394) (11K).

## Discussion

### Effects of Cocaine on the Expression of Opioid Receptors

In the present work, the expression of the zebrafish µ opioid receptor (Fig. 1A) was altered during the different stages of embryonic development. These changes are in agreement with the observations of Azaryan et al. (1998) [46] and Yuferov et al. (1999) [47], who found changes in MOR after cocaine administration to rats. Since the analgesia produced by morphine is mainly due to its interaction with the MOR [1], the changes induced by cocaine in MOR expression could induce alterations in its function [14], [48] and therefore in its analgesic activity.

In regard to DORs (Fig. 1B and C), *zfdor1* showed higher mRNA expression in comparison to *zfdor2* during several stages of the embryonic development and also *zfdor1* (Fig. 1B) showed more marked changes than *zfdor2* (Fig. 1C) after cocaine exposure. These results could be due to the fact that *zfdor1* and *zfdor2* have different spatial and temporal expression profiles during zebrafish development [49] and also because their pharmacological properties are different [44], even though they share greater homology with each other (71%) [43], [44]. The fact that *zfdor2* showed fewer changes induced by cocaine could be due to its potential role during embryogenesis, since DORs have been related to neurogenesis and neuroprotection [50], [51].

We also determined for the first time the expression of *zfmor, zfdor1* and *zfdor2* (Fig. 6A, G and M, respectively) by *ISH* and qPCR at the end of the gastrulation period in zebrafish embryos. Our findings corroborate the presence and the importance of delta opioid receptors in early human embryogenesis [51]. The novelty of the present study is that the opioid receptors studied were distributed in the future cephalic structures along the entire embryonic axis, the posterior trunk and the tail bud region, indicating that all three receptors and the interactions among them can mediate different and crucial actions during the early embryonic development in zebrafish. Our *ISH* and qPCR observations revealed that cocaine induced an increase in the expression of all opioid receptors at 10 hpf, suggesting a potential role of cocaine in the alterations seen in early embryogenesis by acting through the opioid receptors.

Our previous studies using oligoprobes revealed the presence of opioid receptors during zebrafish embryonic development [49], but oligoprobes are much less sensitive than riboprobes. *zfmor*, *zfdor1* and *zfdor2* transcripts were distributed in a similar way at 24 hpf (in the telencephalon, diencephalon, optic tectum, midbrain, midbrain-hindbrain boundary, hindbrain, spinal cord and eye). The expression pattern at 48 hpf remained similar to that observed at 24 hpf (with the addition of the medial longitudinal fascicle, notochord, somites, heart and pectoral flipper). The expression of the opioid receptors in the CNS and peripheral areas were similar to those found in other animal models, e.g., mammals (human, rat and mouse) [52], and likewise to the spatial distribution of the opioid receptors in zebrafish [44], [49], [53] observed upon employing oligoprobes.

Accordingly, taking into consideration the spatial and temporal expression, together with the changes produced by exposure to cocaine in opioid receptors observed in this study, we provide evidence about the expression of the opioid receptors during zebrafish embryonic development, suggesting that an early alteration of opioid receptor expression by cocaine could have repercussions in the various processes in which opioid receptors are involved: the development of the CNS [54], neurogenesis, neuroprotection [50] and its modulation [55], the modulation of neuronal survival [56], vascular development [57], the modulation of the cells of the immune system [58], heart development [59], and a putative role in the development of the neuromuscular system and its function(s) [49].

### Cocaine and *dre-let-7d* miRNA Expression

*dre-let-7d* was expressed in the CNS and at the periphery, which is in agreement with the findings obtained with qPCR and *ISH* in mammals showing that let-7d and its precursors are expressed in the CNS (in the ventral tegmental area, caudate putamen region, nucleus accumbens, prefrontal cortex and hippocampus) [21]. Likewise, let-7 family members have been reported to be present in embryonic tissues, in neural precursors, and also in the developing nervous system of the zebrafish [60], [61]. In the present study, cocaine exposure to the embryos induced an increase at 24 hpf and a decrease at 48 hpf on the expression of opioid receptors; besides, opioid receptors overexpression at 48 hpf reduced the expression of the precursor *dre-let-7d-1*. This might appear contradictory since after cocaine exposure (which induced increase of opioid receptors expression) at 24 hpf, we expect a reduction in *dre-let-7d-1*. This could be due to the fact that cocaine has different actions in both precursors *dre-let-7d-1* and *dre-let-7d-2* (Fig. 2B and C, respectively) as well as in the mature miRNA *dre-let-7d* (Fig. 2A). Another possible explanation could be that *dre-let-7d* and its precursors have different sensitivity to the cocaine effects. It is evident (Fig. 2B), that *dre-let-7d-1* increased its expression (statistically significant) by cocaine exposure at 24 hpf. However, this change was not enough to produce a significant increase in the expression of the mature miRNA. Likewise, the increase in *dre-let-7d-2* expression was correlated with the increase in *dre-let-7d* expression, suggesting that cocaine affects the expression of miRNA-precursors and could induce changes in the expression of the mature miRNA [16], [62], [63]. In this sense, our observations seem to confirm that cocaine increases the expression of proteins involved in miRNA biogenesis: Drosha, Dicer and Ago2 (Fig. 4A, B and C). This indicates that cocaine altering the expression of proteins related to miRNA biogenesis will have consequences in the expression and the activity of mature miRNAs. Since the enzymes related to the miRNA biogenesis, Drosha, Dicer and Ago2, participate in the formation of several miRNAs, suggest that cocaine effects on the expression of opioid receptors changes are not only due to the effect of let-7d, but also to the activity of other miRNAs. In this sense, it is known that different exogenous agents can induce the increase or decrease of specific miRNAs, for instance, cocaine administration alters the expression of many miRNAs (miR-1, miR-124, miR-181a, miR-29b, miR-31, miR-382, miR-212 and let-7d) in brain regions related to cocaine addiction (nucleus accumbens, ventral tegmental area, prefrontal cortex and dorsal striatum) [21], [64], [65]. Fentanyl downregulates miR-190 in hippocampal neuron cultures [17], [18] and morphine decreases miRNAs such as miR-28, miR-125b, miR-150, and miR-382 in monocytes [19]. These observations suggest that changes on the expression of the different miRNAs have certain specificity that could be due to the involvement of drugs or to the diverse location of different miRNAs in the organism.

The expression of the *dre-let-7d* in the CNS (Fig. 4) suggest that this miRNA has an important role during the formation of the CNS (24 hpf) and primary organogenesis (48 hpf) in zebrafish embryos. Therefore, the changes observed in the expression of *dre-let-7d* miRNA and its precursors produced by cocaine could affect important processes occurring during embryogenesis and the development of the CNS [60], [61] in zebrafish.

### Knockdown and Overexpression of Opioid Receptors Alter the Expression of *dre-let-7d* and its Precursors

Let-7 miRNA is a putative regulator of µ opioid receptor binding in the CDS [21] and within 3′UTR elements of mRNA [23], which can affect the expression of opioid receptors. Our study demonstrated that knockdown of the opioid receptors gene, downregulated *dre-let-7d* and its precursors, *dre-let-7d-1* and *dre-let-7d-2* (Fig. 5). Since Let-7 family is highly conserved across species in both sequence and function [23], we suggest that in zebrafish the down regulation of the dre-let-7d target genes (opioid receptors: ZfMOR, ZfDOR1 and ZfDOR2), also decrease its modulator, the miRNA dre-let-7d. Thus an alteration of one of them will affect the other. Likewise, it is possible that let-7d miRNA in zebrafish is regulating not only the expression of the µ opioid receptor, but also the expression of δ opioid receptors. This indicates that opioid receptors are possible potential targets of *dre-let-7d* in zebrafish.

Furthermore, in our hands the overexpression of *zfmor* and *zfdor1* induced changes in the expression of *dre-let-7d* (Fig. 3J) and its precursor *dre-let-7d-2* (Fig. 3C and G). It is unclear how microinjection of *zfdor1,* which increased the expression of *dre-let-7d-2* (Fig. 3G), conversely decreased *dre-let-7d* (Fig. 3J).

The silencing and overexpression of opioid receptors affected the expression of dre-let and its precursors, and the similar expression of opioid receptors and let-7d miRNA in the CNS and at the periphery indicates that they have a close relationship. A consequence of this interrelationship between opioid receptors and *dre-let-7d* is that miRNAs can downregulate the expression of the MOR and DOR receptors that are involved in the pain process [1], [2], [9], [66], suggesting a potential role of miRNAs in pain.

To rule out the possibility that the changes observed in the expression of opioid receptors and *dre-let-7d* might be due to the effect of cocaine on the proliferative [67], [68] and apoptotic [69], [70] processes, we studied proliferation and apoptosis. Our data showed that cocaine induced changes in the expression of the opioid receptors, *dre-let-7d* and its precursors, independent of their actions in proliferation and apoptosis (Fig. 11).

In the present work we provide, at 48 hpf of zebrafish development, a schematic process (Fig. 12A) that hypothesize how cocaine regulate both the opioid receptors and let-7d levels. We believe that cocaine, by increasing the expression of proteins related to miRNA biogenesis (Drosha, Dicer and Ago2) affects the expression of *dre-let-7d* and its precursors (*dre-let-7d-1* and *dre-let-7d-2*). In this way, an increase of the mature miRNA, *dre-let-7d* would regulate negatively the expression of opioid receptors (ZfMOR, ZfDOR1 and ZfDOR2). We propose that cocaine would downregulate *zfmor, zfdor1 and zfdor2* via dre-let-7d, since overexpression and knockdowns of opioid receptors (ZfMOR, ZfDOR1 and ZfDOR2) altered the expression of *dre-let-7d* and its precursors (Fig. 12B) at 48 hpf of zebrafish development.

In conclusion, the silencing and overexpression of opioid receptors affect the expression of *dre-let-7d* and its precursors, indicating a close relationship between them in zebrafish. Additionally, the effects of cocaine during early embryogenesis of this organism may have repercussions in the different processes in which *dre-let-7d* and opioid receptors are involved.
